# Ultrasound-assisted synthesis of rGO/SiO2-based nanosheets and their electrochemical performances in Li-ion batteries

**DOI:** 10.55730/1300-0527.3554

**Published:** 2023-03-07

**Authors:** Ersu LÖKÇÜ, Süleyman CAN, Mustafa ANIK

**Affiliations:** 1 Department of Metallurgical and Materials Engineering, Eskişehir Osmangazi University, Eskişehir, Turkey; 2 Department of Metallurgical and Materials Engineering, Bilecik Şeyh Edebali University, Bilecik, Turkey

**Keywords:** Graphene oxide, SiO_2_ nanosheet, ultrasonication, Li-ion batteries

## Abstract

In this study, a facile approach has been developed to fabricate GO/SiO_2_ nanosheets by the hydrolysis of tetraethyl orthosilicate (TEOS) in the graphene oxide (GO) solution with the assistance of the ultrasonication. The morphology and structure of the SiO_2_/GO nanosheets were characterized by scanning electron microscopy (SEM), X-ray diffraction (XRD), Fourier transform infrared (FTIR) and Raman spectroscopy. The results showed that the covalently bonded SiO_2_ nanoparticles onto the GO sheets were dense and uniform. The agglomeration of the nanosheets was prevented by the ultrasonication and the layer sizes decreased throughout the synthesis process. The size and thickness of the SiO_2_ nanoparticles were determined by the initially and externally added TEOS amounts, respectively, on the GO surface. The anode performance of the thermally reduced rGO/SiO_2_ nanosheets was also observed in the Li-ion half-cell. The reversible capacity of the synthesized TrGOSN-1.5 anode was 424 mA h g^−1^ at a current density of 100 mA g^−1^.

## 1. Introduction

An extensive effort is being put into the studies related with getting effective useful energy from the renewable energy sources that all these studies, without exception, point out the importance of the energy storage technologies. Lithium-ion batteries (LIBs) have taken place at the heart of the commercial energy storage technologies due to their high power and energy densities [1, 2]. However, commercial LIBs, which contain graphite (372 mA h g^−1^) anodes, are insufficient to meet the demands of the developing modern technologies. Many studies conducted on the LIBs have been devoted to the exploration of the alternative high-capacity anode materials to potentially develop the high-performance LIBs. Silicon (Si), silicon monoxide (SiO), and silicon dioxide (SiO_2_) have been attracted great interest as the alternative anode materials for the LIBs due to their high theoretical capacities (Si = 4600 mA h g^−1^, SiO = 2600 mA h g^−1^, SiO_2_ = 1965 mA h g^−1^), low operating potentials (vs. Li/Li^+^), earth-abundances and good environmental compatible properties [3–5]. Their huge volume changes (Si = 300%, SiO = 150%, SiO_2_ = 100%) upon charging and poor conductivities, however, result in capacity fading during the cycling process [5, 6]. Therefore, many studies have focused on synthesizing of the carbon-based Si/SiO/SiO_2_ nanocomposites, which have enhanced conductivity and volume change tolerance [7–10].

Among the nanocomposites, GO/SiO_2_ nanostrucures are used very commonly as a template for the synthesis of Si/graphene nanostructures [11], SiO_2_/Si nanosheets [12], and porous graphene architectures [13] by applying various reduction and etching process combinations. Firstly, Gao and Kou reported a simple one-step solution approach without using any surfactants to synthesize GO/SiO_2_ nanostructures [14]. SiO_2_ nanoparticles (approximately 50 nm) densely form onto the GO layers by the nucleation and growth process and then they fully cover the GO layer. As the alternative route, Singh et al. prepared GO/SiO_2_ nanocomposites by mixing separately synthesized GO and SiO_2_ nanoparticles using the ultrasonic liquid processor [15]. Obviously, according to the many reported studies [14–19], GO/SiO_2_ nanostructures can be synthesized by two main strategies. The first pathway is the addition of the separately synthesized SiO_2_ nanoparticles into the GO solution and then mixing of them by ultrasonically and/or mechanically with or without surfactants [15, 17]. The advantages of this two-step method are controllable distribution and size of the SiO_2_ nanoparticles in the GO/SiO_2_ nanostructures. However, this method is time-consuming and complicated because of the surface modification process of the separately synthesized SiO_2_ nanoparticles. The second strategy is the one-step method that includes the hydrolysis of TEOS in the presence of GO [14, 18]. Although this strategy is relatively simple and fast in preparation of the GO/SiO_2_ nanostructures, there are still challenges in the distribution and size adjustments of the SiO_2_ nanoparticles.

In the present work, GO/SiO_2_ nanosheets (GOSNs) were synthesized by the hydrolysis of TEOS in the presence of GO with the aid of ultrasound-assisted method. The effects of additional TEOS content and ultrasonication process were also investigated on the morphology of GO/SiO_2_ nanosheets. Furthermore, the obtained GOSNs were converted into the rGO/SiO_2_ nanosheets (TrGOSNs) and the morphology and the effect of SiO_2_ loading on the electrochemical performances of electrodes were evaluated for the LIBs.

## 2. Experimental details

### 2.1. Preparation of GO

GO was synthesized by an improved method, which has been reported by our previous study [9]. According to this method, the mixture of acids, H_2_SO_4_:H_3_PO_4_ (360:40 mL), was slowly added into a mixture of KMnO_4_ (18.0 g) and graphite flakes (3.0 g). The reaction mixture was then heated to 50 **°C and stirred at 300 rpm for 12 h. The reaction mixture was cooled down to room temperature normally and poured onto ice (400 mL) at which time H**_2_O_2_ (3 mL) was added dropwise into the mixture. After this step, the mixture was filtered and washed. The initial washing step was performed with 30% HCl solution and it was repeated until getting the transparent supernatant. Then the washing process was continued with DI water and ethanol until achieving a neutral pH value. All washing process was performed by centrifugation at 6000 rpm for 30 min. The resulting solid was dispersed in DI water by ultrasonication at a concentration of 1.0 mg mL^−1^.

### 2.2. Preparation of GO/SiO_2_ and rGO/SiO_2_ nanosheets

GO/SiO_2_ nanosheets (GOSNs) were synthesized by the hydrolysis of TEOS in the GO medium. In detail, GO dispersion (50 mL), ethanol (50 mL), and hydrous ammonia (3.75 mL) were mixed and stirred for 10 min to obtain a homogeneous suspension. Subsequently, TEOS (3.0 mL) was added into the suspension by the peristaltic pump with a rate of 0.225 μL min^−1^. The suspension was stirred for 150 min and then sonicated by the Sonics VCX 750 ultrasonic processor for 30 min. The resulting sample was designated as GOSN-3.0 according to the initial TEOS amount. Further experiments were conducted in order to investigate the effect of initial and additional TEOS amount on the morphologies of GOSNs. Starting chemicals and their amounts are given in Table. Moreover, a schematic flowchart illustrating the steps and optical image of the experimental set-up for the synthesis of GOSNs are provided in Figure S1.

The as-synthesized GOSNs were centrifuged and washed 3 times with DI water and ethanol, then dried at room temperature. Next, GOSNs samples were reduced at 800 **°**C for 4 h under Ar atmosphere to obtain rGO/SiO_2_ nanosheets (TrGOSNs).

### 2.3. Structural characterization

The surface morphologies of the synthesized samples were characterized by SEM (ZEISS Ultraplus). The crystal structure properties of samples were investigated by XRD (PANalytical Empyrean). FT-IR and Raman spectra were obtained using PerkinElmer Spectrum Two and Renishaw Raman InVia Microscope spectrometers, respectively. Thermogravimetric analysis of the samples was carried out using a PerkinElmer Diamond TG/DTA instrument under nitrogen with a heating rate of 5 °C min^−1^.

### 2.4. Electrochemical characterization

The TrGOSN-based anodes were prepared by mixing 75 wt% active material, 15 wt% carbon black (Super P), and 10 wt% polyvinylidene fluoride (PVDF) in N-methyl pyrrolidinone (NMP) to form a homogeneous slurry. Next, the slurry was coated onto the copper foil and dried in a vacuum oven at 80 °C for 12 h. The coated copper foil was then cut into disc electrodes with a diameter of 16 mm. The average mass of the electrodes (SuperP, PVDF binder and active material) was calculated as 0.8 mg, 1.2 mg, 1.2 mg, and 1.3 mg for the TrGOSN-1.5, TrGOSN-3.0, TrGOSN-4.5, and TrGOSN-6.0 samples, respectively. The capacity values were calculated based on the average mass of the electrodes. The CR2016 type coin cell was assembled in an argon-filled glove box with H_2_O and O_2_ levels less than 0.1 ppm. Lithium metal was used as the counter and reference electrodes and the glass microfiber filter as a separator. 1 M lithium hexafluorophosphate (LiPF_6_) in ethylene carbonate and dimethyl carbonate (EC/DMC) in a 1:1 ratio by volume was used as the electrolyte. The charge-discharge tests were performed galvanostatically in a potential range between 0.01 V and 2.50 V (vs Li/Li^+^) at various current densities. Electrochemical impedance spectroscopy (EIS) measurements were performed in the frequency range changes from 1 Hz to 100,000 Hz with an amplitude of 5 mV.

## 3. Results and discussion

**Figure 1:**
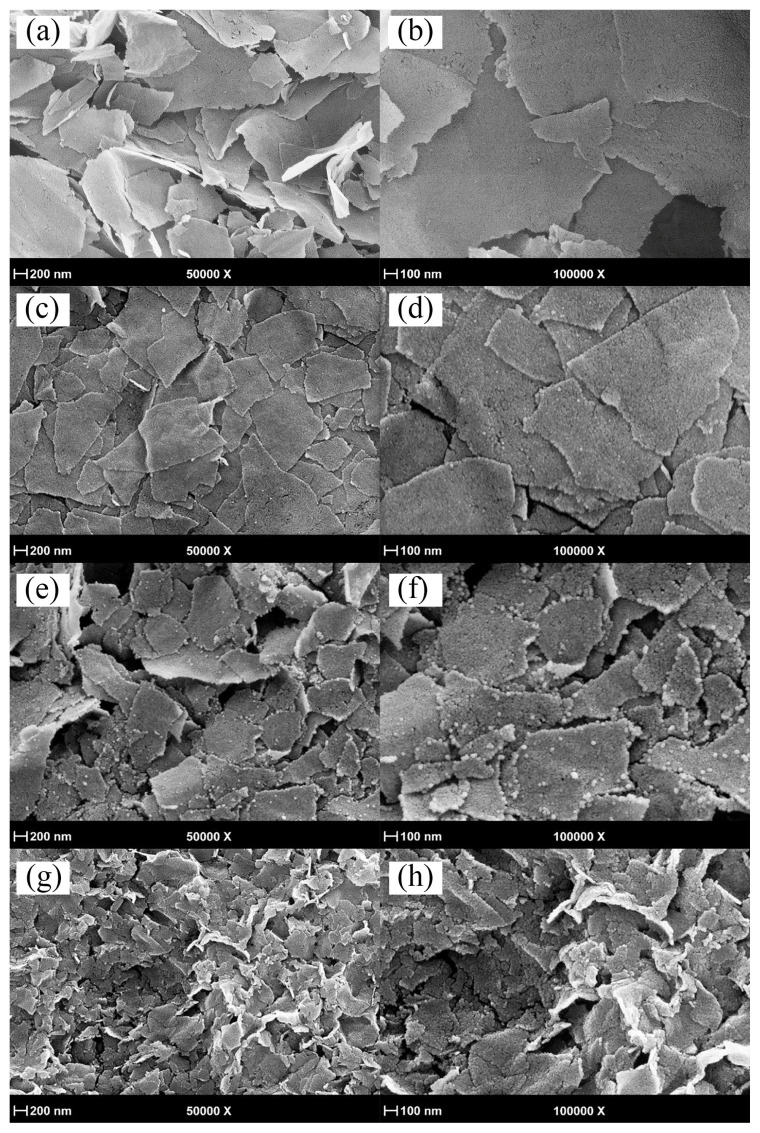
The SEM images of (a, b) GOSN-1.5, (c, d) GOSN-3.0, (e, f) GOSN-4.5*, and (g, h) GOSN-6.0* samples at various magnifications.

The SEM images of graphite used in the synthesis process and those of synthesized GO are given in Figure S2. While the graphite surface has the flat structure, GO shows thin sheet morphology with the wrinkled and rough surfaces arising from the oxidation. Figure 1 shows the SEM images of the as-prepared GOSNs samples by the ultrasound-assisted synthesis method at various magnifications. The surfaces of the GO sheets are uniformly and densely covered with SiO_2_ nanoparticles. It is observable that the sizes of the sheets decrease and the new SiO_2_ nanoparticles form on GO surfaces by extending the ultrasonication process and adding the extra TEOS. The SEM images point out that these SiO_2_ nanoparticles nucleate initially on the edges of the broken GOSNs and then spread onto the surface of the preformed GOSNs. As seen in the SEM picture, the sheets become thicker thanks to the newly formed SiO_2_ nanoparticles. In addition, the SEM images of the synthesized GOSN-1.5 (Figures 1a and 1b) and GOSN-3.0 (Figures 1c and 1d) samples clearly indicate the effect of the initial TEOS amount on the sheet morphology. The low amount of TEOS enables the formation of smaller SiO_2_ nanoparticles onto the GO surface.

**Figure 2:**
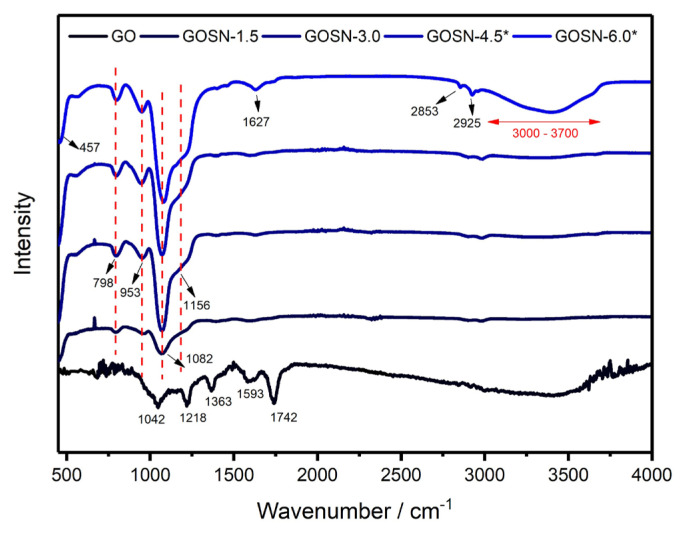
The FT-IR spectra of GO and GOSNs samples.

Figure 2 shows the FTIR spectra of GO and GOSNs samples. The typical FT-IR spectra of GO sample in Figure 2 are in good agreement with the results available in the literature [18, 20]. The broad peak placed between 3700 cm^−1^ and 3000 cm^−1^ originates from the stretching vibrations of O-H bond. The peaks at 1742 cm^−1^ and 1593 cm^−1^ correspond to the C=O stretching of carboxylic and/or carbonyl groups and the C=C sp^2^-hybridization, respectively. The peak at 1363 cm^−1^ is related with the C-OH group while the peaks at 1218 cm^−1^ and 1042 cm^−1^ arise from C-O-C and C-O groups, respectively, in the GO structure. For the GOSNs sample, the peaks at 1082 cm^−1^ and 457 cm^−1^ originate from the asymmetric and bending vibrations of Si-O-Si bonds, respectively. In addition, the symmetric vibrations of Si-O-Si bonds create a peak at 798 cm^−1^.

The shoulder peak at 1156 cm^−1^ is related to both transverse and longitudinal optical modes of the asymmetric vibrations of Si-O-Si/Si-O-C bonds [21, 22]. The peak located at 953 cm^−1^ is due to the stretching vibration of Si-OH bonds. The reduction in the intensity of the C=O peak at 1742 cm^−1^ can be attributed to the conversion of C=O bonds into the Si-O-C bonds [21, 22]. This conversion implies the presence of covalent linkage between GO surface and SiO_2_ nanoparticles. As clearly seen from Figure 2, the functional groups on the surfaces of all the synthesized samples are similar. However, there is an increase in the infrared wave absorption peak associated with the Si-O–based groups depending on the increase in the SiO_2_ loading. This result is not surprising since the movement freedom of the Si-O functional groups gradually increases on the sample surface [22, 23].

**Figure 3:**
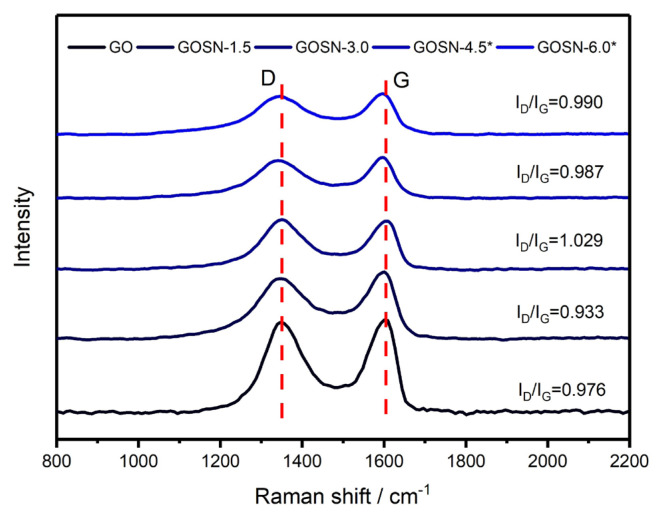
The Raman spectra of GO and GOSNs samples.

Figure 3 shows the Raman spectra of GO and GOSNs samples in the range changing from 800 cm^−1^ to 2200 cm^−1^. In the spectra, the characteristic D-band and G-band peaks of carbon materials are clearly observable [14, 24]. The D-band represents the disordered or sp^3^-hybridized carbons, while the G-band represents the vibrations of sp^2^-hybridized carbon atoms in the structure. For the GO sample, the D-band and G-band are located at 1348 cm^−1^ and 1604 cm^−1^, respectively. After introducing the SiO_2_ nanoparticles onto the GO surface, the D-band and G-band peak positions shift slightly to 1340 cm^−1^ and 1598 cm^−1^, respectively. This shifting is probably due to the attachments of the SiO_2_ nanoparticles onto the GO surface [14, 15]. In addition, the D-band to G-band intensity ratios (I_D_/I_G_) of the GO and GOSNs samples are given as inset in Figure 3. During the synthesis, the attachment of the SiO_2_ nanoparticles onto the GO surface and the ongoing sonication process cause the I_D_/I_G_ ratio to increase slightly.

The formation of the SiO_2_ nanoparticles on the GO layers is also confirmed by energy-dispersive X-ray spectroscopy (EDX), XRD, and TGA analysis for selected GOSN sample. The results are given in supplementary materials. It can be seen from the EDX pattern (Figure S3b) that the as-synthesized GOSN-3.0 sample shows sharp Si peak (40.27 wt%), O peak (44.58 wt%), and relatively low intensity C peak (15.15 wt%) without any impurity. The high amount Si and O peaks are expected since the EDX analysis is a surface sensitive. The XRD pattern of the as-synthesized GOSN-3.0 sample is given with graphite and GO patterns in Figure S3a. The GOSN-3.0 sample shows only a broad diffraction peak at 2θ = 22.72° and it does not show any diffraction peak at 2θ = 10.54°, which belongs to the GO, due to the dominant effect of amorphous SiO_2_ [20, 25].

**Figure 4:**
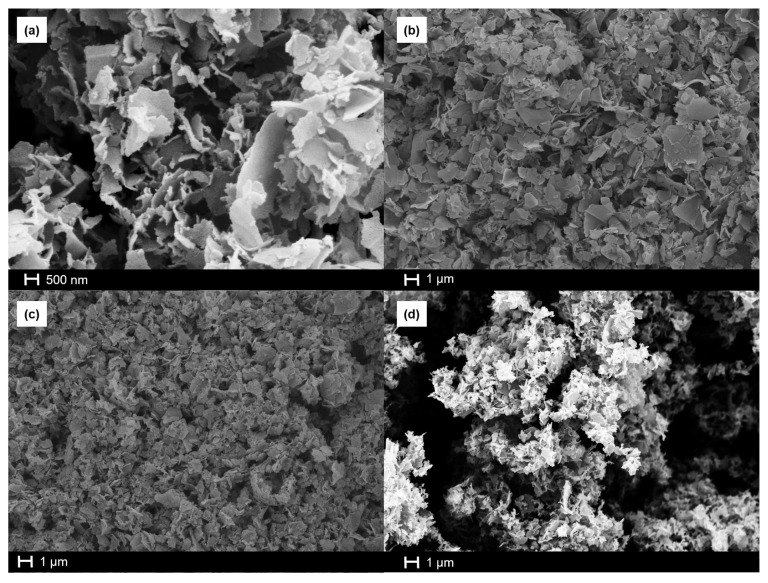
The SEM images of (a) TrGOSN-1.5, (b) TrGOSN-3.0, (c) TrGOSN-4.5, and (d) TrGOSN-6.0 samples.

After the thermal reduction, the morphological and structural changes from GOSNs to TrGOSNs were observed by SEM, FT-IR, and Raman spectroscopy techniques. Figure 4 shows a SEM image of TrGOSN samples. As clearly seen from this image, the samples keep their sheet morphology without noticeable agglomeration due to the thermal reduction process.

**Figure 5:**
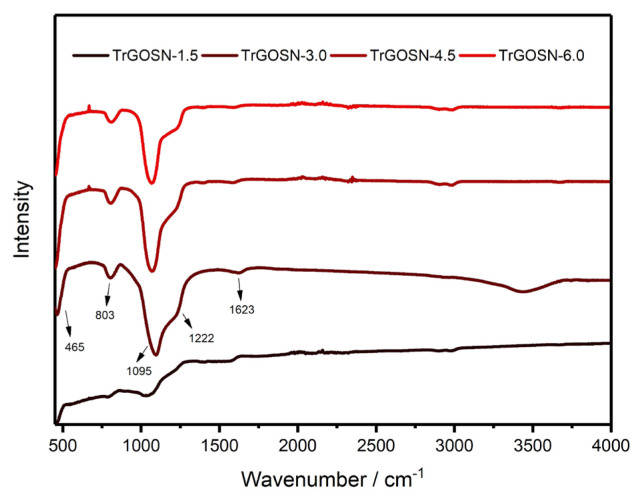
The FT-IR spectra of GO and TrGOSNs samples.

FT-IR spectra of TrGOSN samples are shown in Figure 5. The most visible change upon the thermal reduction of GOSNs samples is the disappearance of the peak located at 953 cm^−1^, which corresponds to the stretching vibration of Si-OH bonds. This change is due to the hydrolytic polycondensation process of the SiO_2_ nanoparticles [26]. The sharp adsorption peak at 1095 cm^−1^ is ascribed to the Si-O-Si and Si-O-C stretching vibrations. The stretching and bending vibrations of Si-O bonds are represented by the peaks at 803 cm^−1^ and 465 cm^−1^, respectively. On the other hand, the FT-IR spectra of TrGOSN samples seem to be weaker and flatter as compared to those of GOSNs samples since they form as a result of not only the GO reduction, but also the reaction between functional groups and Si-OH. Despite all these reactions, the Si-O-C covalent bonding between rGO sheets and SiO_2_ nanoparticles is kept firmly [21–23].

**Figure 6:**
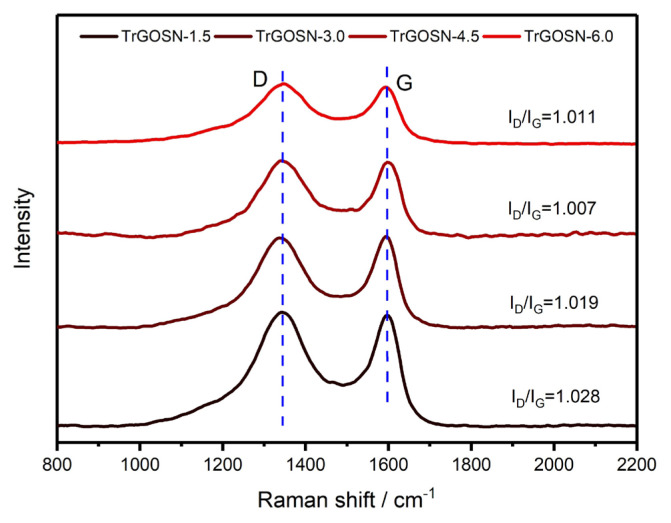
The Raman spectra of GO and TrGOSNs samples.

The Raman spectra of TrGOSNs samples are shown in Figure 6. The D and G bands appear at around 1344 cm^−1^ and 1596 cm^−1^, respectively, for all the samples. While there is no significant shift in the D and G bands, the I_D_/I_G_ intensities are close to each other. The I_D_/I_G_ ratio reflects the degree of irregularity and it is a criterion for the reduction of GO to rGO [27]. Compared to the GO (I_D_/I_G_ = 0.976) and GOSNs (I_D_/I_G_ below approximately 1.000) samples, TrGOSNs samples have higher I_D_/I_G_ ratios (>1.000), which show the formation of more sp^2^ hybridization, indicative of the successful reduction.

**Figure 7:**
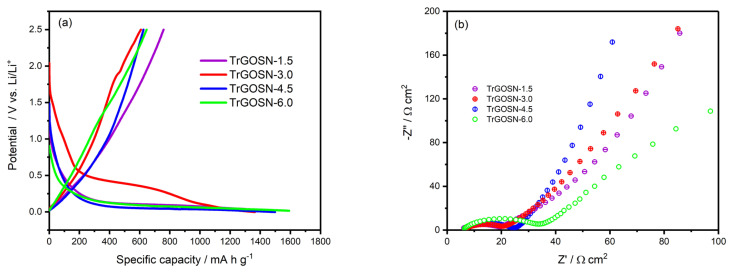
(a) The discharge/charge curves of the TrGOSN electrodes at 100 mA g^−1^, (b) Nyquist plots of the TrGOSN electrodes before the electrochemical tests.

The galvanostatically measured initial discharge/charge profiles of the TrGOSN electrodes at a current density of 100 mA g^−1^ are provided in Figure 7. The initial discharge capacities of the TrGOSN-1.5, TrGOSN-3.0, TrGOSN-4.5 and TrGOSN-6.0 electrodes are 1307 mA h g^−1^, 1364 mA h g^−1^, 1498 mA h g^−1^, and 1581 mA h g^−1^, respectively. With the increase in the SiO_2_ content of the electrodes, the discharge capacities also increase, but as it is seen from the charge curves, the initial coulombic efficiencies (CEs) of the electrodes are quite low. The electrodes cannot maintain their discharge capacities in the subsequent cycles and suffer a sudden capacity loss.

This irreversible capacity, after the first cycle, arises from the formation of the SEI layer and the irreversible lithiation reactions of SiO_2_ as they can be written in Eqs. (1) and (2) [28, 29]. The irreversible characteristics of the formed Li_2_O and Li_4_SiO_4_ products lead to the consumption of the Li^+^ ions and thus cause the capacity loss in the initial cycles. On the other hand, the elemental Si formed in Eqs. (1) and (2) reacts with Li^+^ ions as depicted in Eq. (3), which causes the battery to work reversibly [29, 30].


(1) $${\text{SiO}}_{2}+4{\text{Li}}^{+}+4{\text{e}}^{-}\to 2{\text{LI}}_{2}\text{O}+\text{Si},$$

(2) $$2{\text{SiO}}_{2}+4{\text{Li}}^{+}+4{\text{e}}^{-}\to {\text{Li}}_{2}{\text{SiO}}_{4}+\text{Si},$$

(3) $${\text{Si}}_{2}+{\text{xLi}}^{+}+{\text{xe}}^{-}↔{\text{Li}}_{\text{x}}\text{Si}.$$

The initial CEs of the TrGOSN-1.5, TrGOSN-3.0, TrGOSN-4.5, and TrGOSN-6.0 electrodes are 57.94%, 45.55%, 41.72%, and 40.89%, respectively. These CEs increase up to 98.0% after the 20th cycle. The relatively higher initial CE value of the TrGOSN-1.5 electrode can be attributed to its SiO_2_ content. Considering the above equations, the initial CE values are low for other electrodes, since the presence of more SiO_2_ results in consumption of more Li^+^ ions during the initial charge/discharge process [31, 32]. Figure 7b shows the EIS spectra of the fresh electrodes at the open circuit potentials. The EIS analysis is conducted to determine the conductivity and charge-transfer properties of the as-prepared electrodes. The charge transfer resistance (R_ct_) values at the electrode/electrolyte interface are calculated as 18.7 Ω cm^2^, 19.2 Ω cm^2^, 26.4 Ω cm^2^, and 38.9 Ω cm^2^ for the TrGOSN-1.5, TrGOSN-3.0, TrGOSN-4.5, and TrGOSN-6.0 electrodes, respectively. The charge transfer resistances increase slightly depending on the SiO_2_ content.

**Figure 8:**
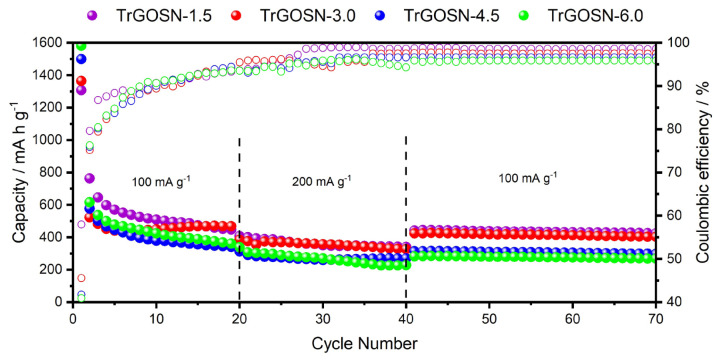
The cycling performance and coulombic efficiencies of the TrGOSN electrodes.

The cycling and rate performances of TrGOSNs electrodes are shown in Figure 8 for the cycle range changes from 1st to 70th. While the capacity of TrGOSN electrode decreases during the initial cycles, it starts to stabilize gradually in the following cycles. This behavior is probably due to the continuation of the irreversible reactions and the ongoing activation process of TrGOSNs electrodes [28–30]. At the end of the 20th cycle, the capacities of TrGOSN-1.5, TrGOSN-3.0, TrGOSN-4.5, and TrGOSN-6.0 electrodes are 446 mA h g^−1^, 468 mA h g^−1^, 350 mA h g^−1^, and 361 mA h g^−1^, respectively. When the discharge current densities increase up to 200 mA g^−1^, the capacities of TrGOSN-4.5 and TrGOSN-6.0 continue to decrease, while those of TrGOSN-1.5 and TrGOSN-3.0 are relatively more stable. The TrGOSN-1.5, TrGOSN-3.0, TrGOSN-4.5, and TrGOSN-6.0 electrodes deliver the reversible capacities of 424 mA h g^−1^, 408 mA h g^−1^, 297 mA h g^−1^, and 268 mA h g^−1^, respectively, at the end of the 70th cycle when the discharge current density returns back to 100 mA g^−1^. The obtained results of the electrochemical characterizations show that the cycling performance of TrGOSN-1.5 electrode, which has small size SiO_2_ with lesser SiO_2_ loading, is quite satisfactory. Designing of a study includes synthesis of these layers with different SiO_2_ sizes and loadings can be suggested to obtain the improved electrochemical performance.

## 4. Conclusion

In summary, GO/SiO_2_ nanosheets were successfully prepared by the ultrasound-assisted synthesis method. The effects of different amounts of TEOS and ultrasonication process on the morphology of GO/SiO_2_ nanosheets were also observed. The ultrasonication prevented the agglomeration of the nanosheets and caused the layer sizes to decrease throughout the synthesis process. While the initial TEOS amount determined the size of SiO_2_ nanoparticles, the addition of extra TEOS during the synthesis process did not change the size of the SiO_2_ nanoparticles. The addition of extra TEOS during the synthesis, however, increased the thickness of the SiO_2_ layer formed on the GO layer. By transforming GO/SiO_2_ nanosheets into the thermally reduced SiO_2_/GO nanosheets, the initial and stable anode capacities were measured as 1307 mA h g^−1^ and 424 mA h g^−1^, respectively, in Li-ion half-cell for the TrGOSN-1.5 sample, which has the lower SiO_2_ content and smaller nanoparticles. Thus, we believe that this work provides the new insights into the SiO_2_-based nanosheet materials not only for the energy applications but also for the various other applications.

## Supplementary materials

Figure S4 shows the TGA curves of GO and GOSN-3.0 samples. The initial weight loss of up to 150 °C is attributed to the removal of the physically adsorbed water molecules from both samples. In the temperature changes from 150 °C to 800 °C, the GOSN-3.0 sample exhibits negligible weight loss, while the GO loses 40% of its initial weight. The removal of the chemical water from the unstable carboxylic and hydroxyl groups accompanied to the release of CO_2_ as a result of the decomposition of carboxylic groups causes a sharp weight loss between 150 °C and 30 °C for the GO sample [33]. The weight loss observed between 300 °C and 800 °C is due to the removal of more stable oxygen groups such as carbonyl from the GO structure [33, 34]. The results of the TGA analysis indicate that SiO_2_ nanoparticles attached to the GO surface improve the thermal stability of the GO.

Figure S1 The flowchart illustrating the steps and optical image of the experimental set-up for the synthesis of GOSNs.

Figure S2 SEM images of (a) graphite and (b) graphene oxide.

Figure S3 (a) The XRD patterns of graphite, GO, GOSN-3.0, and TrGOSN-3.0 sample, **(b)** EDX analysis of GOSN-3.0 sample.

Figure S4 The TGA curves of GO and GOSN-3.0 samples.

## Figures and Tables

**Table:** The starting amounts of the chemicals used in the synthesis of GOSN samples.

Samples	GO (1 mg mL^−1^)	Ethanol	Hydrous ammonia	TEOS	H_2_O	Total time
GOSN-1.5	50 mL	50 mL	3.75 mL	1.5 mL		3 h
GOSN-3.0	50 mL	50 mL	3.75 mL	3.0 mL	-	3 h
GOSN-4.5*	-	-	-	additional 1.5 mL	0.55 mL	6 h
GOSN-6.0*	-	-	-	additional 1.5 mL	0.55 mL	9 h
