# Supplementary figures and images for: Ultrasound-assisted synthesis of rGO/SiO2-based nanosheets and their electrochemical performances in Li-ion batteries
Source: Turk J Chem. 2023 Mar 7;47(2):495–503. doi: 10.55730/1300-0527.3554 (PMC10388086; doi:10.55730/1300-0527.3554)

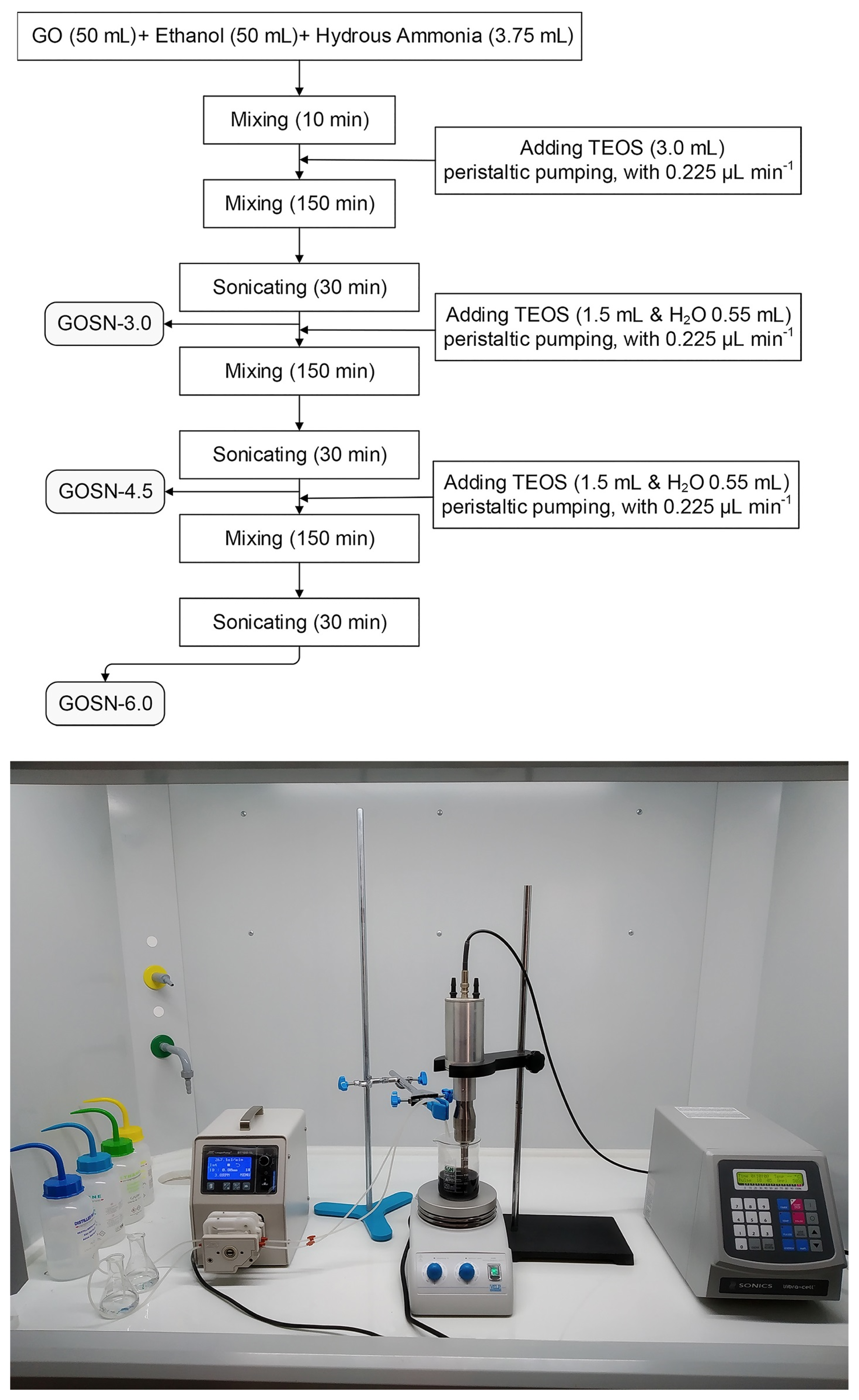

Supplement: Figure S1 — The flowchart illustrating the steps and optical image of the experimental set-up for the synthesis of GOSNs. [file turkjchem-47-2-495s1.tif]

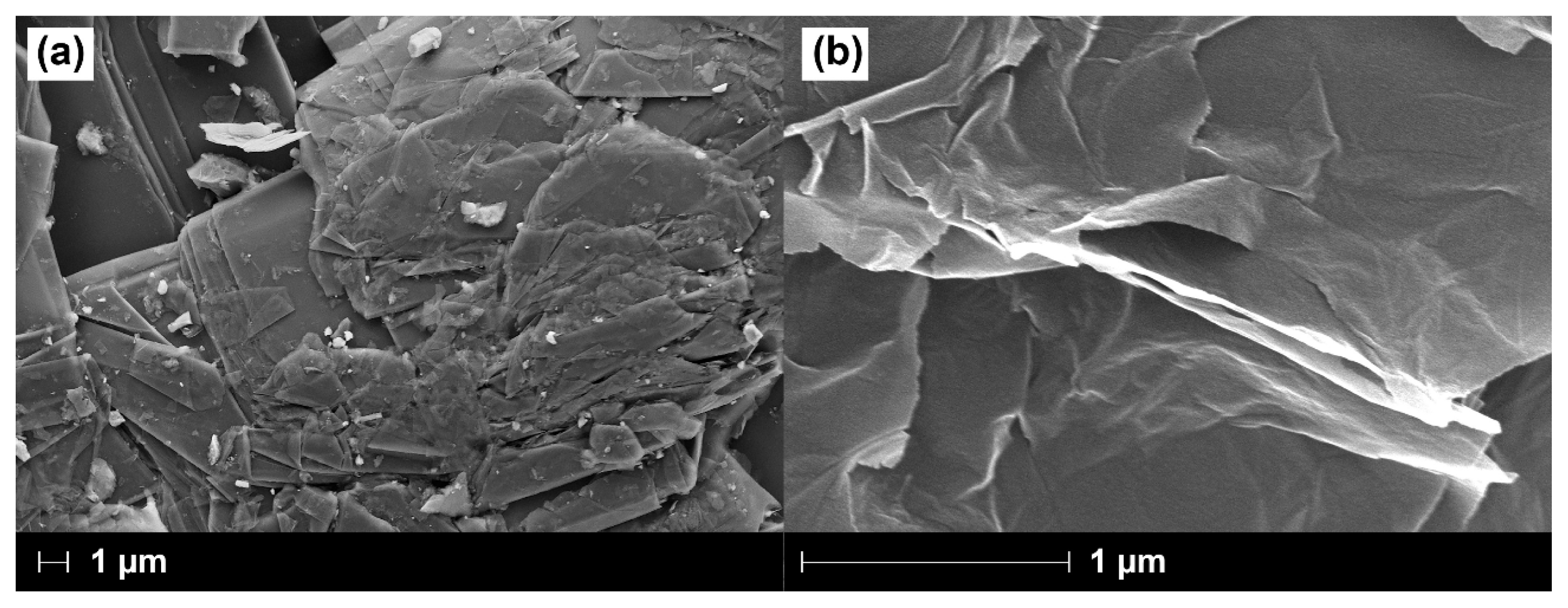

Supplement: Figure S2 — SEM images of (a) graphite and (b) graphene oxide. [file turkjchem-47-2-495s2.tif]

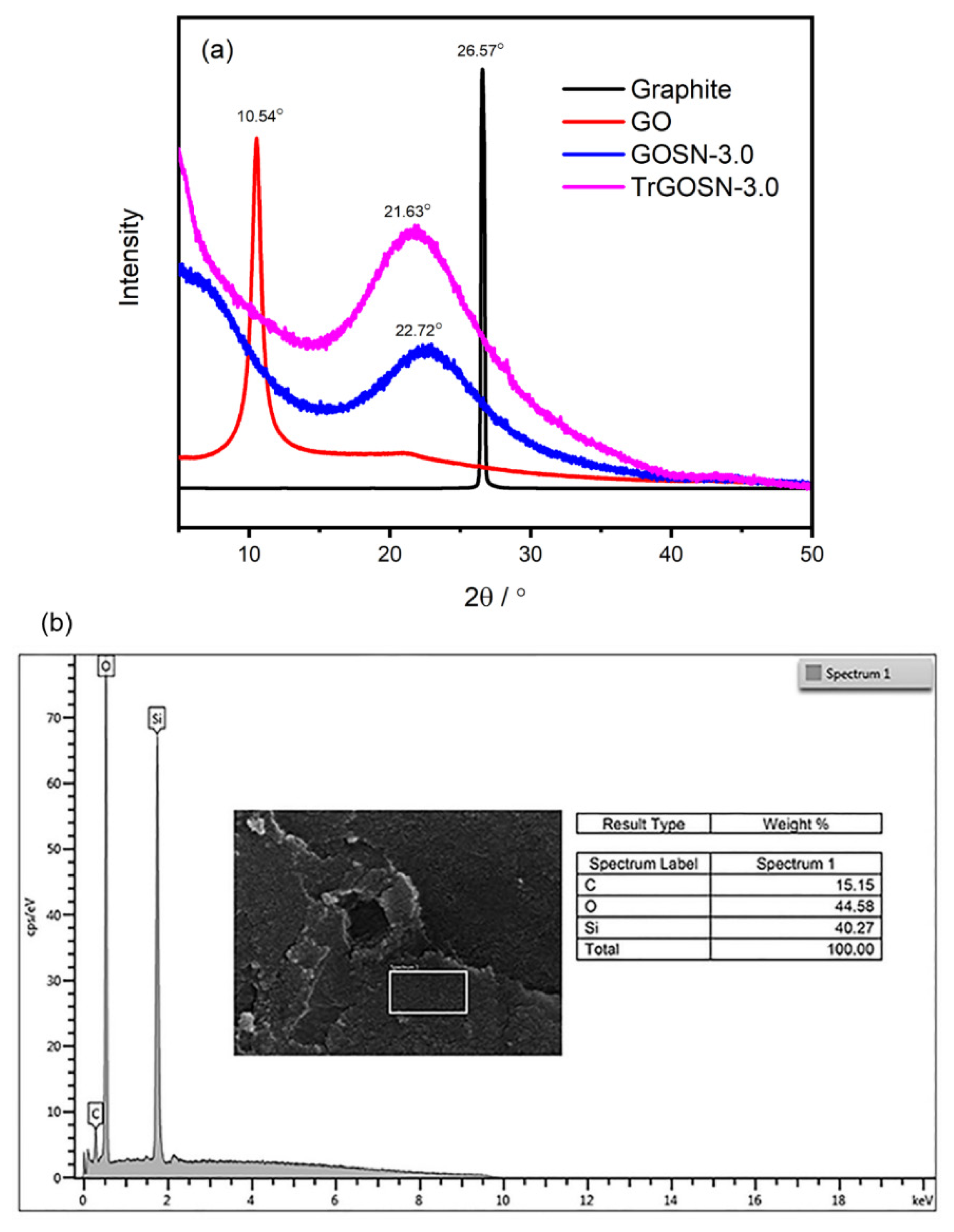

Supplement: Figure S3 — (a) The XRD patterns of graphite, GO, GOSN-3.0, and TrGOSN-3.0 sample, (b) EDX analysis of GOSN-3.0 sample. [file turkjchem-47-2-495s3.tif]

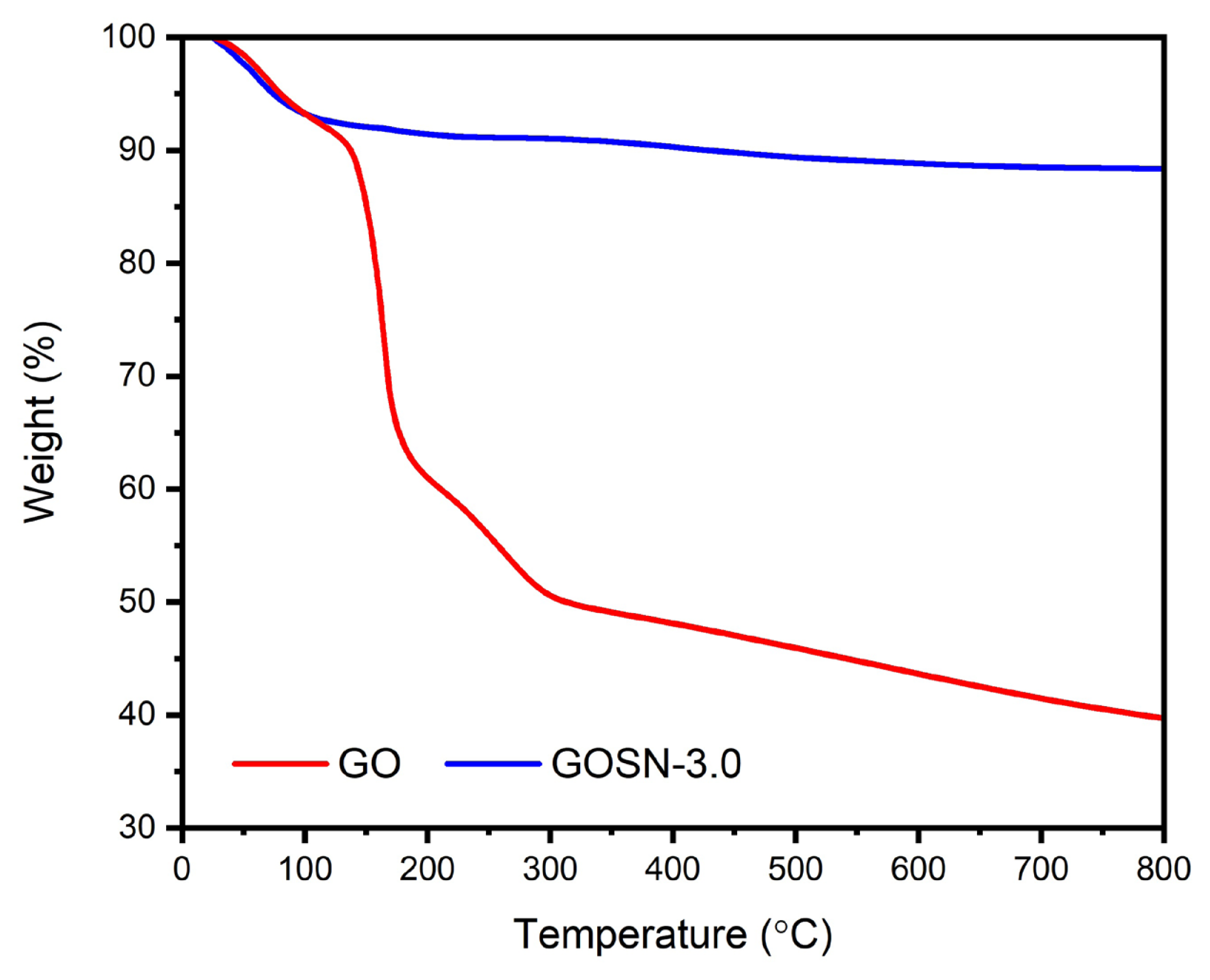

Supplement: Figure S4 — The TGA curves of GO and GOSN-3.0 samples. [file turkjchem-47-2-495s4.tif]
